# Assessment of the Efficacy of a Low-Dose Iron Supplement in Restoring Iron Levels to Normal Range among Healthy Premenopausal Women with Iron Deficiency without Anemia

**DOI:** 10.3390/nu15112620

**Published:** 2023-06-03

**Authors:** Matthew W. Stefan, David M. Gundermann, Matthew H. Sharp, Brooke A. Jennings, Raad H. Gheith, Ryan P. Lowery, Tieraona LowDog, Somsuvra B. Ghatak, Jose Barbosa, Jacob M. Wilson

**Affiliations:** 1 The Applied Science and Performance Institute, Research Division, Tampa, FL 33607, USA; mstefan@theaspi.com (M.W.S.); msharp@theaspi.com (M.H.S.); bjennings@theaspi.com (B.A.J.); rgheith@theaspi.com (R.H.G.); rlowery@theaspi.com (R.P.L.); 2 MegaFood, Manchester, NH 03103, USA; david.gundermann@megafood.com (D.M.G.); som.ghatak@megafood.com (S.B.G.); jose.barbosa@megafood.com (J.B.); 3 Medicine Lodge Ranch, Pecos, NM 87552, USA; lowdogmd@drlowdog.com

**Keywords:** low iron, iron supplement, Women’s Health, gastrointestinal, constipation, ferritin, iron

## Abstract

(1) Background: Iron deficiency without anemia (IDWA) is a prevalent health concern in premenopausal women. Oral supplementation of iron may be a viable solution to improve blood-iron status in women; however, the effects of a high-dose iron-supplement regimen have been associated with gastrointestinal side effects. Therefore, the purpose of the present study was to evaluate the effectiveness of a low-dose liquid fermented iron-bisglycinate supplement (LIS) on improving blood-iron status in premenopausal women with IDWA without increasing constipation or gastrointestinal distress. (2) Methods: 85 premenopausal women with IDWA (ferritin < 70 ng/dL and hemoglobin > 11.0 g/dL) took a LIS (27 mg) or a placebo (PLA) for 8 weeks. Blood draws were taken at Wk0 and Wk8 of the study to measure serum-iron markers. In addition, surveys of gastrointestinal distress were administered at Wk0, Wk4, and Wk8 while the profile of mood states (POMS) was surveyed at Wk0 and Wk8. (3) Results: Compared to the placebo, the LIS was able to increase serum ferritin (*p* = 0.03), total serum iron (*p* = 0.03), and mean corpuscular volume (*p* = 0.02), while exhibiting no significant interaction in subjective gastrointestinal distress (*p* > 0.05). No significant effects were detected for POMS (*p* > 0.05). (4) Conclusions: Supplementing with LIS appears to improve blood-iron status without causing significant gastrointestinal distress in premenopausal women with IDWA.

## 1. Introduction

Iron deficiency anemia currently affects 1.2 billion people globally [1]. While iron deficiency anemia is a commonly recognized and diagnosed medical condition, iron deficiency without anemia (IDWA) is a common mineral deficiency that affects approximately 1.6 billion of the world’s population, and despite its prevalence, is not easily recognized nor diagnosed [2]. Iron deficiency with anemia is diagnosed once hemoglobin values fall below a threshold value (i.e., <11.0 g/dL); however, symptoms of iron deficiency may be present before that occurs. Therefore, monitoring other markers of iron status, such as ferritin, may be a more optimal indicator of iron stores and a specific biomarker for assessing general iron deficiency [2,3]. Recent models define iron deficiency in at least 3 stages, including latent (<70 ng/mL of ferritin, reduced iron, but normal hemoglobin), pre-anemic (<30 ng/mL of ferritin, reduced hemoglobin and absent iron staining), and anemic (<15 ng/mL of ferritin, absence of iron staining, and hemoglobin <11 g/dL, and absent iron in bone marrow) with symptoms beginning to appear in the latent stage [3,4,5,6,7,8]. Thus, for the present study, IDWA was defined by ferritin values <70 ng/mL, and a hemoglobin value >11.0 g/dL [1,3,9,10].

Iron deficiency in women may stem from a variety of causes: inadequate dietary intake, increased iron need for the body, impaired iron absorption, chronic inflammation, and/or blood loss [2]. Iron deficiency is most prevalent in women of childbearing age, accounting for approximately 70% of the global iron-deficient population [11]. Women are at risk due to an increased need for iron during pregnancy and repetitive blood loss during menstruation. In addition, women tend to consume fewer calories than men, which may lead to an inadequate dietary intake of some essential vitamins and/or minerals [12]. Therefore, increasing iron intake through supplementation may be a viable solution in women to prevent iron deficiency with or without anemia. However, the available literature on iron supplementation primarily investigates high-dose iron intake for the treatment of iron deficiency anemia (IDA). These studies typically involve a dosage range of 60–200 mg/d [13,14], and this range is associated with an increased incidence of unwanted gastrointestinal and constipation side effects [15]. Furthermore, it is evident that the form of iron also plays a role in efficacy and incidence of side effects from supplementation. For example, iron chelates compared to iron salts are associated with improved efficacy and gastrointestinal symptoms [16,17,18]. Thus, it can be presumed that a low-dose iron chelate may be a desirable alternative to a higher-dose iron salt in terms of both efficacy and safety. However, it is widely unknown how effective the recommended daily allowance (RDA) of an iron chelate can be in supporting the iron levels of healthy women with IDWA. The Institute of Medicine of the National Academies declares that the highest RDA for iron, accommodating healthy premenopausal female populations, including during pregnancy, is 27 mg of elemental iron while the tolerable upper limit is 45 mg [19]. This research was intended to evaluate the effectiveness of an iron dietary supplement as an alternative to a prescription iron treatment.

Thus, the aim of this study was to evaluate the efficacy and tolerability of a low-dose (27 mg) liquid fermented iron-bisglycinate supplement (LIS) in healthy premenopausal women with IDWA. Changes in various iron markers in blood were monitored to determine the efficacy of the supplement. The study also examined subjective gastrointestinal effects using the Patient Assessment of Constipation Symptoms (PAC-SYM), and the Gastrointestinal Symptoms Rating Scale (GSRS) was used to examine the frequency and severity of overall gastrointestinal discomfort, such as nausea, vomiting, acid reflux, cramping, and black stools, experienced by the study participants. Finally, the abbreviated Profile of Mood States (POMS) questionnaire was administered to assess subjective mood and energy levels. Our hypothesis was that the LIS would result in an improvement in blood-iron status without significantly increasing constipation or gastrointestinal distress compared to the placebo.

## 2. Materials and Methods

### 2.1. Study Design

This 8-week intervention study was carried out in a randomized, double-blind, placebo-controlled, parallel manner. All participants were screened for eligibility according to the inclusion and exclusion criteria (Supplementary File) and were randomized into one of two groups: placebo (PLA) or LIS. The study required three study visits: baseline (Wk0), midpoint (Wk4), and final testing (Wk8). At Wk0, participants underwent standard sterile venipuncture to provide a blood sample for hematological variables and completed all study questionnaires (PAC-SYM, GSRS, and POMS). At Wk4, participants completed the PAC-SYM and GSRS only. Testing at Wk8 was conducted in an identical manner to Wk0. Supplement compliance and adverse events were assessed at Wk4 and Wk8. Hematological variables were considered as primary outcomes while questionnaires and adverse events were considered as secondary outcomes. For all study visits, participants reported to the investigative study site (Applied Science and Performance Institute; Tampa, FL, USA) to complete the study procedures, which are further described below.

### 2.2. Participants

**Figure 1:**
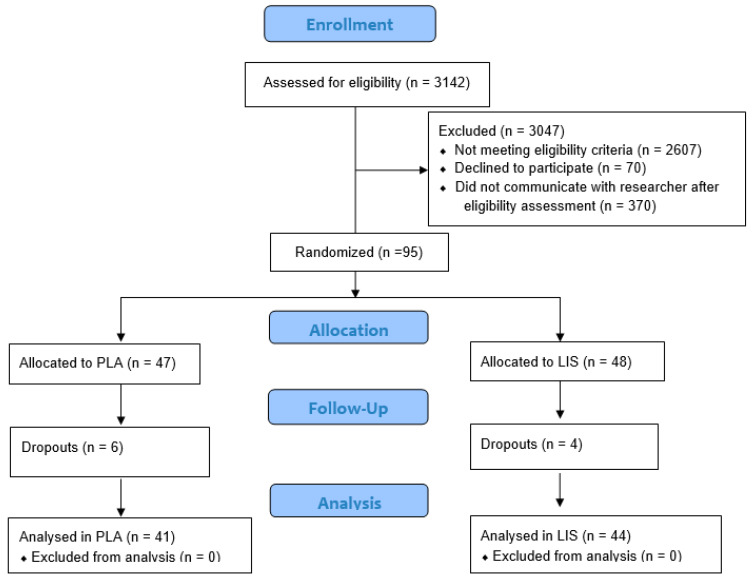
CONSORT Diagram.

To determine the sample size for the study, an *a priori* power analysis (G*Power, version 3.0.10) was performed. According to a systematic review and meta-analysis of 28 randomized controlled trials with a total of 1493 participants investigating supplemental iron tablets [20], the standardized mean difference in blood-iron status between the supplemental iron tablet and placebo group was 0.63. Using a two-tailed, paired t-test with the input parameters set as flowed; effect size = 0.63, alpha = 0.05, and power = 0.80 (80%); the resulting sample size was 41 participants per group or 82 total. To account for potential dropouts, the enrollment target goal was increased by approximately 20% over the total sample size determined by the power analysis. Participants were recruited from the Tampa Bay area via word of mouth, email contact, and digital recruitment services. A total of 3142 potential female participants completed an eligibility screening questionnaire (Figure 1). A total of 3047 were excluded from participation for not meeting eligibility criteria (2607), declining to participate (70), or failing to communicate with research staff following eligibility screening (370). A total of 95 premenopausal female participants were enrolled in the study based on the inclusion and exclusion criteria. These 95 participants were quartile ranked according to their serum-ferritin levels and participants forming each quartile were randomly assigned to a study group in a 1:1 ratio (PLA n = 47, LIS n = 48) via randomizer.org (Social Psychology Network, Middletown, CT, USA) in a blind manner by a staff member of the investigative site who was not involved in study data collection. Enrolled participants were assigned a unique study number in the 100 series as the subject ID for the study to maintain anonymity. At the end of the study period, the PLA group contained 41 participants, and the LIS group contained 44 participants. There were no significant differences between groups for BMI, systolic blood pressure, diastolic blood pressure, heart rate, and oxygen saturation (*p* > 0.05). Table 1 provides the baseline characteristics of the 85 participants who completed the study. The 10 participants who did not complete the study failed to attend follow-up testing due to time constraints, work, or family responsibilities. Prior to engaging in any study procedures, participants signed a written informed consent for participation. The protocol was approved by an external Institutional Review Board (Advarra; Columbia, MD, USA; Protocol #Pro00060191) and registered at clinicaltrials.gov (ID: NCT05257343).

### 2.3. Supplement Protocol, Blinding, and Compliance

The LIS was composed of the following: 30 total calories, 7 g of carbohydrates, 10 mcg of vitamin B12 (cyanocobalamin), 27 mg of iron (as fermented iron bisglycinate), and other ingredients including organic glycerin, water, apple juice concentrate, pear juice concentrate, tart cherry juice concentrate, natural flavors, beetroot concentrate, and citrus peel extract. The PLA was composed of the same formula as the LIS condition except for 27 mg of iron (fermented iron bisglycinate) and vitamin B12 (cyanocobalamin). Products for both study conditions were produced and provided by MegaFood (Manchester, NH, USA) in visually identical bottles with a label on each bottle containing a blind study-condition code, dose instructions, net quantity of product, lot number, and expiration date. The participants and study investigative staff were blind to the study treatment. A single non-investigative staff member was not blind to the study treatment in case any serious adverse events occurred requiring a description of the study treatment for medical purposes.

Upon enrollment into the study, participants were provided with two bottles of their respective condition (either PLA or LIS). The researchers weighed each bottle provided to the participant. Participants were also provided with two small measuring cups so that a 10 mL dose could be self-dispensed and self-administered by the participant. When participants returned for a check-in visit at Wk4, they were provided with one additional bottle. Each participant received a total of 3 bottles for the entire enrollment period (8 weeks). Participants were instructed to consume one serving (10 mL) of the supplement per day, at least three hours removed from their last meal.

Upon return to the research facility at Wk4, the researcher collected the bottles, weighed them, and either discarded the empty bottles or returned the unfinished bottles to the participants. Once participants returned for their final Wk8 visit, the researchers collected any remaining bottles the participant had and weighed them. The differences in weight between each visit were calculated, which was then divided by the number of days that the participant was supplementing to determine how much weight by volume the participants consumed over the course of the study to assess compliance. The average compliance rate for the PLA and LIS groups over the course of the entire study was 90.11% and 90.80%, respectively.

### 2.4. Blood Sampling

At Wk0 and Wk8, participants were asked to fast overnight for at least 10 h prior to undergoing a blood draw. Venous blood was extracted via standard sterile venipuncture of the antecubital vein by a certified phlebotomist using a 21-gauge syringe and collected into 2 vials (1) an 8.5 mL gel-barrier, marble top tube interiorly coated with silicone (BD Vacutainer^®^, SST™, Becton, Dickinson and Company, Franklin Lakes, NJ, USA) and (2) a 5 mL lavender top EDTA vacutainer tube (BD Vacutainer^®^, Becton, Dickinson and Company, Franklin Lakes, NJ, USA). Afterward, the gel-barrier tubes were inverted 6–8 times and allowed to clot for approximately 30 min at 4 °C. Gel-barrier tubes were then centrifuged at 1665 g for 15 min at 4 °C and the resulting serum samples were aliquoted and stored at −80 °C until the sample could be analyzed for an iron panel, which included iron, total iron binding capacity, transferrin saturation percentage, and ferritin. Serum samples were thawed once and analyzed in duplicate in the same assay for each analysis to avoid compounded inter-assay variance. Whole-blood samples, collected into the lavender top EDTA vacutainers tubes, were inverted 6–8 times, stored at 4 °C, and analyzed within 3 days of collection for a standard complete blood-count analysis. All samples were sent to a local Labcorp (Laboratory Corporation of America Holdings, Burlington, NC, USA) for analysis.

### 2.5. Gastrointestinal Symptom Rating Scale (GSRS)

Participants were instructed to complete the Gastrointestinal Symptom Rating Scale (GSRS) at Wk0, Wk4, and Wk8. The GSRS is a subjective instrument of 15 items combined into the following 5 symptom domains: acid reflux, abdominal pain, indigestion, diarrhea, and constipation. A 7-point Likert-type scale was used to grade the GSRS where 1 represents the absence of troublesome symptoms and 7 represents very troublesome symptoms [21].

### 2.6. Patient Assessment of Constipation-Symptoms (PAC-SYM)

Participants were instructed to complete the Patient Assessment of Constipation-Symptoms (PAC-SYM) at Wk0, Wk4, and Wk8. The PAC-SYM questionnaire was used as a tool for assessing the severity of patient-reported symptoms of chronic constipation. The 12-item questionnaire is divided into 3 symptom subscales: abdominal (4 items); rectal (3 items); and stool (5 items). Items are scored on 5-point Likert scales, with scores ranging from 0 to 4 (0 = symptom absent, 1 = mild, 2 = moderate, 3 = severe, and 4 = very severe). A mean total score in the range of 0–4 was generated by dividing the total score by the number of questions completed; the lower the total score, the lower the symptom burden [22].

### 2.7. Abbreviated Profile of Mood States (POMS)

Participants were instructed to complete the abbreviated Profile of Mood States (POMS) at Wk0 and Wk8. The abbreviated POMS used in this study is a 40-item version where participants rate each item on a 5-point Likert scale with anchors ranging between “Not at all” to “Extremely”. Items are combined to form 6 separate subscales: tension, depression, anger, vigor, fatigue, and confusion. The subscale scores are then combined to form an overall measure of affect that is labeled as total mood disturbance (TMD). A lower score indicates lower mood disturbance while a higher score indicates increased mood disturbance [23].

### 2.8. Adverse Events

Researchers asked the participants if they had experienced any adverse events on each visit in terms of incidences of bloating, diarrhea, heartburn, nausea, constipation, upset stomach, headache, and/or abdominal discomfort. In addition, participants were instructed to report any adverse events immediately to the researcher via email to begin the documentation process. The frequency of an adverse event was defined as how many participants reported no adverse effects (0), reported an adverse event one time over the course of the study (1), or if the adverse event was reported twice by a participant over the course of the study (2). Severity was defined as follows: no adverse event (0), the adverse event was mild (1), the adverse event was moderately severe (2), or the adverse event was severe (3).

### 2.9. Statistical Analysis

All statistical analyses were carried out at the completion of the study using GraphPad Prism (Version 9; San Diego, CA, USA). Data were assessed for normality and equal variance assumptions via Shapiro-Wilk and Levene’s test, respectively, prior to executing any inferential statistics. When assumptions were not met, non-parametric alternatives (Mann-Whitney, Wilcoxon, and Friedman’s test) were used. Statistical significance of primary and secondary outcomes was assessed with a two-way mixed model analysis of variance (ANOVA) assuming group (PLA and LIS) as the between-participants factor, time (Wk0 and Wk8) as the within-participants factor, and participants as a random factor. Whenever a significant F-value was obtained, a post hoc test with Bonferroni’s adjustment was applied for multiple comparisons. Statistical significance was set at *p* < 0.05 for all analyses. Effect sizes were calculated as Cohen’s *d* for between-participants designs as the difference between group means divided by the pooled standard deviation times a correction factor [24]. Data are reported as mean ± standard deviation.

$$\mathrm{Cohen}’s\;d=\frac{\left(X_{F2,WK8}-X_{F2,WK0}\right)-\left(X_{M2,WK8}-X_{M2,WK0}\right)}{SDpooled}*(1-\frac{3}{4\times \left(N1+N2\right)–9})$$

## 3. Results

### 3.1. Hematological Outcomes

#### 3.1.1. Blood-Iron Status

**Figure 2:**
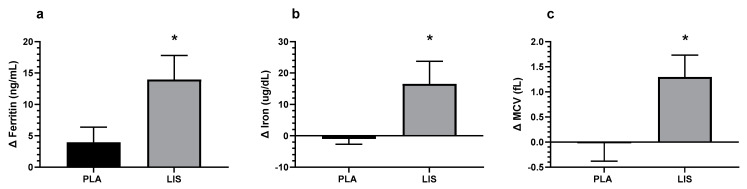
Delta change in ferritin concentration (**a**), Blood-iron concentration (**b**), and mean corpuscular volume (MCV) (**c**). * = Significantly greater than PLA (*p* < 0.05). Data is the mean and standard error of the mean.

A significant group-by-time interaction was detected for ferritin indicating that the change demonstrated from Wk0 to Wk8 was significantly different between groups (*p* = 0.033, LIS: +13.96, PLA: 3.95, Mean Diff: +10.01, 95% CI: 0.83 to 19.17 μg/dL; Figure 2a). Post hoc analysis indicated that only the LIS group demonstrated greater ferritin concentration at Wk8 compared to Wk0 (*p* < 0.001, +49.0%, Mean Diff = +13.95, 95% CI = 6.65 to 21.26 ng/mL; Table 2).

A significant main time effect was detected for total iron-binding capacity (TIBC) whereby values at Wk8 were significantly lower than Wk0 (*p* = 0.001, Mean Diff = −11.56, 95% CI = −18.45 to −4.67 μg/dL; Table 2). A significant group-by-time interaction was detected for total serum iron concentration indicating that the change demonstrated from Wk0 to Wk8 was significantly different between groups (*p* = 0.030, LIS: +16.5, PLA: −4.63, Mean Diff: +21.13, 95% CI: 2.06 to 40.21 μg/dL; Figure 2b). Post hoc analysis indicated that the blood-iron concentration in the LIS group was significantly higher at Wk8 compared to the PLA group (*p* = 0.041, +20.3%, Mean Diff = +20.44, 95% CI = 0.71 to 40.16 μg/dL; Table 2). While no significant interaction or main effects were detected for transferrin saturation (tSAT) (*p* > 0.05), it was trending towards a significant interaction effect (*p* = 0.067; Table 2). The raw mean and standard deviation can be found in Table 2.

#### 3.1.2. Complete Blood Count

A significant group-by-time interaction was detected for mean corpuscular volume (MCV), indicating that the change demonstrated from Wk0 to Wk8 was significantly different between groups (*p* = 0.022, LIS: +1.30, PLA: −0.02, Mean Diff: +1.32, 95% CI: 0.19 to 2.45 fL; Figure 2c). Post hoc analysis indicated that only the LIS group demonstrated greater MCV at Wk8 compared to Wk0 (*p* = 0.003, +1.5%, Mean Diff: +1.30, 95% CI: 0.40 to 2.19 fL; Table 3).

A significant main time effect was detected for red blood cells (RBC) (*p* = 0.021, Mean Diff = +0.08, 95% CI: 0.01 to 0.15 M/uL), hemoglobin (*p* < 0.001, Mean Diff = +0.39, 95% CI = 0.22 to 0.56 g/dL), hematocrit (*p* < 0.001, Mean Diff = +1.04, 95% CI: 0.48 to 1.61%), and monocytes (%) (*p* = 0.048, Mean Diff = −0.39, 95% CI: −0.79 to −0.01%; Table 3). No significant interactions or main effects were detected for the remainder of the variables included in the complete blood count panel (*p* > 0.05, Table 3).

### 3.2. Gastrointestinal Symptom Rating Scale (GSRS) and the Patient Assessment of Constipation-Symptoms Questionnaire (PAC-SYM)

Data for the Gastrointestinal Symptom Rating Scale (GSRS) and the Patient Assessment of Constipation-Symptoms Questionnaire (PAC-SYM) failed normality testing; therefore, non-parametric statistics were deployed. For the GSRS, significant within-group changes were detected for the Total GSRS Score in the PLA group (Friedman *p* = 0.008) in which Wk4 was higher than Wk8 (*p* = 0.02; Table 4). For the PAC-SYM, the relative change from Wk0 to Wk8 was significantly different between groups in which the PLA group increased constipation symptomology (PLA = +0.17 a.u., LIS = −0.03 a.u., Mann Whitney *p* = 0.049; Table 4).

### 3.3. Profile of Mood States (POMS)

Data for the Profile of Mood States (POMS) failed normality testing; therefore, non-parametric statistics were deployed. No significant within- or between-group differences were detected for POMS Total Score (*p* > 0.05; Table 5).

### 3.4. Adverse Events

There was a total of 50 adverse events reported throughout the duration of the study. Forty-six of the adverse events were related to the study, while 4 were not related to the study. The adverse events that were not related to the study were a COVID-19 infection, a medical condition unrelated to the study, vaginal dryness, and a urinary infection with hematuria. No serious adverse effects were reported (death, hospitalization, or emergency room visit). Chi-squared analysis revealed no significant differences between PLA and LIS in the perceptions of the frequency or severity of constipation, bloating, nausea, upset stomach, abdominal discomfort, diarrhea, headache, or heartburn (Table 6). 

## 4. Discussion

The study aimed to assess the impact of the administered LIS on iron biomarkers in healthy premenopausal women with IDWA. LIS or PLA was administered daily at a dose of 10 mL for 8 weeks. Baseline measurements (Wk0) and final testing data (Wk8) were collected for the primary outcomes, which included an iron panel and complete blood count values. Secondarily, this study assessed gastrointestinal and constipation symptoms via the GSRS and the PAC-SYM questionnaires, respectively, at Wk0, Wk4, and Wk8. Lastly, the abbreviated POMS was administered at Wk0 and Wk8. The present study is the first to show that the LIS significantly improved total serum iron, ferritin, and MCV in women with IDWA. Additionally, this supplement regimen was not associated with any significant negative effects in gastrointestinal or constipation symptomology surveyed in the GSRS and PAC-SYM. No significant effects were detected for POMS.

It is well established that low ferritin levels are indicative of low body-iron stores and can be associated with negative health effects even in the absence of low hemoglobin levels or iron deficiency anemia [1]. Given the crucial role of iron in human health, including its impact on energy metabolism, oxygen transport, and acid-base balance [25], it is imperative to investigate iron-supplementation strategies in varying deficiency stages. Iron deficiency without anemia affects a substantial portion of the population, particularly premenopausal women [26]. While a formal clinical diagnosis of IDWA has yet to be established, most research concurs that serum ferritin between 15 and 70 ng/mL [3,9,10] in combination with symptoms such as fatigue, weakness, and impaired concentration [1,3] may be indicative of IDWA. The present study adds to our body of knowledge regarding the prevention and treatment of IDWA with a low-dose, highly bioavailable liquid iron supplement that is both effective and well-tolerated [7,9,11,14,20]. 

In addition to increasing ferritin levels, the LIS also demonstrated an improvement in MCV. Improvements in MCV values may indicate that red blood cells are becoming more homogenous in size and volume, thereby improving the overall quality of red blood cells. Monitoring MCV aids in identifying the specific type of anemia: microcytic anemia, macrocytic anemia, or normocytic anemia [27]. Lower values of MCV (microcytic anemia) are an indicator of iron deficiency [1], and this information enables a more comprehensive and accurate assessment of the participants’ blood-iron status to help determine the cause of the abnormal value [27]. Additionally, although there was no statistically significant difference in tSAT between groups (*p* = 0.067), the results showed a trend towards statistical significance, with the LIS demonstrating a non-significant increase (+18.01%), and the PLA demonstrating a decrease (−4.36%) at Wk8 post-supplementation. Participants that exhibit low tSAT levels may experience difficulties in utilizing iron for erythropoiesis; therefore, increasing tSAT provides enough iron for healthy red blood cell production [28].

The secondary objectives of the study included assessing the frequency and severity of gastrointestinal discomfort and/or constipation through gastrointestinal assessments (GSRS and PAC-SYM). The results indicated that supplementation of the LIS group did not lead to significant changes in gastrointestinal discomfort over the 8-week study period. However, oral iron supplementation (usually in the form of tablets or capsules) tends to have low absorption rates, requiring iron doses ranging from 60–200 mg [13]. Previous research has shown that this dosage primarily contributes to the commonly reported gastrointestinal discomfort symptoms associated with iron supplementation [29]. A meta-analysis and systematic review of oral iron ferrous supplementation by Tolkien et al. [15] found that a dosage of <60 mg, led to fewer gastrointestinal side effects than higher dosages. Tolkien et al. [15] suggested that these iron salts lead to gastrointestinal symptoms by iron-induced redox recycling, which then leads to inflammation and alterations in the gut microbiota and their metabolism. Furthermore, previous research corroborates Tolkien et al. [15] by demonstrating that an overabundance of oral iron supplementation has negative effects on the gut microbiome [30,31], and may lead to an increase in inflammation [32,33]. Thus, it would be optimal to use the minimum effective dose to mitigate these adverse effects while simultaneously considering that iron supplements come in various forms, i.e., iron chelates (iron bisglycinate) and iron salts (ferrous iron), with iron chelates demonstrating higher bioavailability than ferrous iron without the previously mentioned gastrointestinal symptoms [16,17,18]. In addition, the recommended daily allowance for iron as declared by the Institute of Medicine of the National Academies for healthy premenopausal female populations is 27 mg of iron per day [19], the dosage used in the iron supplement in this study. Therefore, the use of a more bioavailable form of iron in combination with a lower dosage could explain the minimal gastrointestinal discomfort and improvements in blood iron profiles. 

Low-dose liquid iron supplementation (e.g., 27 mg) appears to be an effective nutritional strategy for restoring blood iron profiles in premenopausal women with IWDA as indicated in the results presented in the current study. To sustain these outcomes, routine check-ups should be performed for the maintenance of blood-iron profiles. Previous research has shown that supplementation can be discontinued once ferritin levels have been corrected with follow-up testing conducted every 6–12 months or as needed to monitor iron status [2]. It would have been valuable to the present study if follow-up blood assessments were collected after cessation of supplementation to assess the long-term effectiveness of the LIS at maintaining improved iron-biomarker values. Finally, future studies could expand the population to include children, who are also susceptible to iron deficiency [34].

In conclusion, the results of this study provide evidence that supplementing with a LIS is effective in improving blood-iron biomarkers, as indicated through increases in iron, ferritin, and MCV, while tSAT values were trending towards significance. Importantly, these improvements were achieved without increasing constipation or gastrointestinal distress over the 8-week study period. This study contributes to the growing body of literature on the benefits of iron supplementation. The results suggest that LIS consumption may be a safe and effective way to address IDWA and improve blood-iron status, which can have significant implications for the overall health and well-being of premenopausal women with IDWA.

## Figures and Tables

**Table 1:** Participant baseline characteristic data.

Variable	Group	N	Mean		
Age (yrs)	PLA	41	34.95	±	8.58
	LIS	44	35.00	±	8.63
	Total	85	35.98	±	8.56
Height (cm)	PLA	41	166.22	±	6.17
	LIS	44	163.11	±	5.66
	Total	85	164.66	±	2.20
Weight (kg)	PLA	41	66.81	±	7.58
	LIS	44	62.76	±	8.27
	Total	85	64.79	±	2.86
Body Mass Index (kg/m^2^)	PLA	41	24.28	±	3.22
	LIS	44	23.65	±	3.28
	Total	85	23.96	±	0.45
Heart Rate (bpm)	PLA	41	66.24	±	9.69
	LIS	44	68.91	±	12.65
	Total	85	67.58	±	1.89
Systolic Blood Pressure	PLA	41	115.42	±	10.26
(mmHg)	LIS	44	114.57	±	10.54
	Total	85	114.99	±	0.60
Diastolic Blood Pressure	PLA	41	71.22	±	7.40
(mmHg)	LIS	44	68.36	±	9.72
	Total	85	69.79	±	2.02

Data are mean and standard deviation.

**Table 2:** Blood Iron Panel.

Ferritin (ng/mL)	Wk0	Wk8	G × T *p*	*d* (95% CI)
PLA	29.49 ± 18.86	33.44 ± 23.03	0.033 *	0.47 (0.04, 0.89)
LIS	28.48 ± 15.75	42.43 ± 23.85		
Iron (ug/dL)	Wk0	Wk8	G × T *p*	*d* (95% CI)
PLA	82.15 ± 38.85	77.51 ± 44.50	0.030 *	0.47 (0.04, 0.90)
LIS	81.29 ± 35.97	97.79 ± 36.94		
TIBC (ug/dL)	Wk0	Wk8	G × T *p*	*d* (95% CI)
PLA	371.68 ± 51.64	360.59 ± 48.49	0.894	−0.03 (−0.45, 0.39)
LIS	351.46 ± 45.01	339.43 ± 43.94		
tSAT (%)	Wk0	Wk8	G × T *p*	*d* (95% CI)
PLA	22.91 ± 12.05	21.91 ± 12.96	0.067	0.40 (−0.03, 0.82)
LIS	23.93 ± 11.61	28.24 ± 10.53		

Wk0 and Wk8 data are mean  ±  SD. *p*-value is from the group-by-time interaction effect. * = indicates statistical significance (*p* < 0.05). *d* = Cohen’s d between-group effect size. 95% CI = 95% confidence interval of the effect size. TIBC: Total Iron-Binding Capacity, tSAT: Transferrin Saturation.

**Table 3:** Complete Blood Count.

WBC (K/uL)	Wk0	Wk8	G × T *p*	*d* (95% CI)
PLA	6.44 ± 1.79	6.28 ± 1.89	0.773	−0.06 (−0.48, 0.36)
LIS	6.44 ± 1.90	6.17 ± 1.28		
RBC (M/uL)	Wk0	Wk8	G × T *p*	*d* (95% CI)
PLA	4.39 ± 0.37	4.47 ± 0.33	0.900	0.02 (−0.40, 0.45)
LIS	4.52 ± 0.49	4.61 ± 0.45		
Hemoglobin (g/dL)	Wk0	Wk8	G × T *p*	*d* (95% CI)
PLA	12.82 ± 1.02	13.07 ± 1.11	0.122	0.34 (−0.09, 0.76)
LIS	12.86 ± 1.26	13.39 ± 1.20		
Hematocrit (%)	Wk0	Wk8	G × T *p*	*d* (95% CI)
PLA	38.86 ± 2.75	39.57 ± 2.60	0.240	0.25 (−0.17, 0.68)
LIS	39.02 ± 3.33	40.40 ± 3.07		
MCV (fL)	Wk0	Wk8	G × T *p*	*d* (95% CI)
PLA	88.73 ± 4.77	88.71 ± 3.91	0.022 *	0.50 (0.07, 0.93)
LIS	86.93 ± 7.56	88.23 ± 6.99		
MCH (pg)	Wk0	Wk8	G × T *p*	*d* (95% CI)
PLA	29.24 ± 1.72	29.30 ± 2.22	0.209	0.27 (−0.15, 0.70)
LIS	28.69 ± 3.31	29.23 ± 2.95		
MCHC (g/dL)	Wk0	Wk8	G × T *p*	*d* (95% CI)
PLA	32.97 ± 0.97	33.07 ± 2.23	0.846	0.04 (−0.38, 0.46)
LIS	32.95 ± 1.35	33.14 ± 1.58		
RDW (%)	Wk0	Wk8	G × T *p*	*d* (95% CI)
PLA	12.90 ± 1.02	12.90 ± 0.92	0.686	0.09 (−0.33, 0.51)
LIS	13.09 ± 1.56	13.17 ± 1.38		
Platelet Count (K/uL)	Wk0	Wk8	G × T *p*	*d* (95% CI)
PLA	271.81 ± 49.51	274.39 ± 52.09	0.33	−0.21 (−0.63, 0.21)
LIS	295.75 ± 71.29	287.82 ± 65.12		
Neutrophils (%)	Wk0	Wk8	G × T *p*	*d* (95% CI)
PLA	56.46 ± 11.97	57.15 ± 9.10	0.817	−0.05 (−0.47, 0.37)
LIS	57.29 ± 10.95	57.50 ± 10.13		
Lymphs (%)	Wk0	Wk8	G × T *p*	*d* (95% CI)
PLA	30.34 ± 8.20	31.24 ± 7.55	0.462	−0.16 (−0.58, 0.26)
LIS	31.86 ± 9.53	31.80 ± 9.30		
Monocytes (%)	Wk0	Wk8	G × T *p*	*d* (95% CI)
PLA	8.15 ± 2.55	7.90 ± 1.95	0.445	−0.16 (−0.59, 0.26)
LIS	7.66 ± 1.77	7.11 ± 1.93		
Eos (%)	Wk0	Wk8	G × T *p*	*d* (95% CI)
PLA	2.63 ± 1.97	2.78 ± 2.15	0.668	0.09 (−0.33, 0.51)
LIS	2.27 ± 1.78	2.55 ± 1.61		
Basos (%)	Wk0	Wk8	G × T *p*	*d* (95% CI)
PLA	0.90 ± 0.37	0.90 ± 0.49	0.267	0.24 (−0.18, 0.66)
LIS	0.86 ± 0.51	0.96 ± 0.43		
Neutrophils (abs)	Wk0	Wk8	G × T *p*	*d* (95% CI)
PLA	3.83 ± 1.52	3.68 ± 1.53	0.830	−0.05 (−0.47, 0.38)
LIS	3.82 ± 1.63	3.61 ± 1.15		
Lymphs (abs)	Wk0	Wk8	G × T *p*	*d* (95% CI)
PLA	1.90 ± 0.51	1.89 ± 0.52	0.736	−0.07 (−0.49, 0.35)
LIS	1.96 ± 0.52	1.91 ± 0.51		
Monocytes (abs)	Wk0	Wk8	G × T *p*	*d* (95% CI)
PLA	0.51 ± 0.18	0.49 ± 0.17	0.435	−0.17 (−0.59, 0.25)
LIS	0.48 ± 0.13	0.44 ± 0.13		
Eos (abs)	Wk0	Wk8	G × T *p*	*d* (95% CI)
PLA	0.16 ± 0.13	0.17 ± 0.15	0.708	0.09 (−0.33, 0.51)
LIS	0.15 ± 0.13	0.16 ± 0.11		
Basos (abs)	Wk0	Wk8	G × T *p*	*d* (95% CI)
PLA	0.04 ± 0.05	0.04 ± 0.05	0.815	−0.04 (−0.46, 0.38)
LIS	0.05 ± 0.05	0.05 ± 0.05		
Immature Granulocytes (%)	Wk0	Wk8	G × T *p*	*d* (95% CI)
PLA	0.05 ± 0.22	0.00 ± 0.00	0.419	0.17 (−0.25, 0.60)
LIS	0.05 ± 0.21	0.05 ± 0.21		
Immature Granulocytes (abs)	Wk0	Wk8	G × T *p*	*d* (95% CI)
PLA	0.00 ± 0.02	0.00 ± 0.00	0.986	−0.06 (−0.49, 0.36)
LIS	0.00 ± 0.02	0.00 ± 0.00		
hs-CRP (mg/L)	Wk0	Wk8	G × T *p*	*d* (95% CI)
PLA	0.96 ± 0.79	1.18 ± 1.47	0.155	−0.31 (−0.73, 0.12)
LIS	1.17 ± 0.97	1.08 ± 0.88		

Wk0 and Wk8 data are mean  ±  SD. *p*-value is from the group-by-time interaction effect. * = indicates statistical significance (*p* < 0.05). *d* = Cohen’s d between-group effect size. 95% CI = 95% confidence interval of the effect size. WBC: white blood cell, RBC: red blood cell, MCV: mean cell volume, MCH: mean cell hemoglobin, MCHC: mean cell hemoglobin concentration, RDW: red cell distribution width, hs-CRP: high-sensitivity C-reactive protein.

**Table 4:** GSRS and PAC-SYM.

GSRS Total Score	Wk0	Wk4	Wk8	Friedman *p*	M.W.Δ4-0	M.W.Δ8-0	*d* (95% CI)
PLA	1.56 ± 0.48	1.45 ± 0.39	1.60 ± 0.45 ^a^	0.008	0.695	0.533	0.11 (−0.31, 0.53)
LIS	1.76 ± 0.57	1.62 ± 0.408	1.74 ± 0.55	0.451			
PAC-SYM Total Score	Wk0	Wk4	Wk8	Friedman *p*	M.W.Δ4-0	M.W.Δ8-0	*d* (95% CI)
PLA	0.47 ± 0.49	0.45 ± 0.42	0.64 ± 0.49	0.123	0.479	0.049 *	−0.37 (−0.80, 0.05)
LIS	0.69 ± 0.60	0.57 ± 0.43	0.66 ± 0.58	0.475			

Wk0, Wk4, and Wk8 data are mean  ±  SD. Friedman *p*-value was used to obtain within-group differences. Mann Whitney *p*-value was used to obtain the between-group differences using the mean delta change at the Wk4 vs. Wk0, and Wk8 vs. Wk0. * = indicates statistical significance (*p* < 0.05). ^a^ = indicates a statistical significance of the within-group changes between the Wk8 and Wk4 timepoint (*p* < 0.05). *d* = Cohen’s d between-group effect size. 95% CI = 95% confidence interval of the effect size.

**Table 5:** Profile of Mood States (POMS).

Total Score	Wk0	Wk8	Wilcoxon *p*	M.W. *p* Δ8-0	*d* (95% CI)
PLA	86.61 ± 12.90	86.42 ± 11.89	0.702	0.429	0.05 (−0.37, 0.48)
LIS	93.50 ± 17.36	94.05 ± 17.33	0.452		

Wk0, Wk4, and Wk8 data are mean  ±  SD. Wilcoxon *p*-value was used to obtain within-group differences. Mann Whitney *p*-value was used to obtain the between-group differences using the mean delta change at Wk8 vs. Wk0. *d* = Cohen’s d between-group effect size. 95% CI = 95% confidence interval of the effect size.

**Table 6:** Adverse Events Analysis.

			Frequency				Severity				
	Group	0	1	2	χ2	Group	0	1	2	3	χ2
Constipation	PLA	37	3	1	0.639	PLA	37	2	1	2	0.182
	LIS	37	6	1		LIS	37	1	6	1	
	Total	74	9	2		Total	74	3	7	3	
Bloating	PLA	41	0	0	0.084	PLA	41	0	0	0	0.118
	LIS	39	4	1		LIS	39	2	2	2	
	Total	80	4	1		Total	80	2	2	2	
Nausea	PLA	36	5	0	0.905	PLA	36	3	2	0	0.466
	LIS	39	5	0		LIS	39	2	1	2	
	Total	75	10	0		Total	75	5	3	2	
Upset Stomach	PLA	39	2	0	0.277	PLA	39	2	0	0	0.161
	LIS	39	5	0		LIS	39	1	3	2	
	Total	78	7	0		Total	78	3	3	2	
Abdominal Discomfort	PLA	39	1	1	0.580	PLA	39	2	0	1	0.246
	LIS	43	1	0		LIS	43	0	1	0	
	Total	82	2	1		Total	82	2	1	1	
Diarrhea	PLA	39	2	0	0.515	PLA	39	0	0	2	0.213
	LIS	43	1	0		LIS	43	0	1	0	
	Total	82	3	0		Total	82	0	1	2	
Headache	PLA	41	0	0	0.167	PLA	41	0	0	0	0.385
	LIS	42	2	0		LIS	42	0	1	1	
	Total	83	2	0		Total	83	0	1	1	
Heartburn	PLA	41	0	0	0.332	PLA	41	0	0	0	0.332
	LIS	43	1	0		LIS	43	1	0	0	
	Total	84	1	0		Total	84	1	0	0	

Frequency = the number of times an adverse event was reported by a participant, defined as 0 = no adverse event reported, 1 = participant reported adverse event once, 2 = participant reported adverse event twice. Severity was defined as 0 = no adverse event reported, 1 = adverse effect was mild, 2 = adverse effect was moderate, 3 = adverse effect was severe.

## Data Availability

Data is available upon reasonable request to the corresponding author.

## Supplementary Materials

The following supporting information can be downloaded at: https://www.mdpi.com/article/10.3390/nu15112620/s1, Supplementary File: Study Eligibility Criteria.
