# Supplementary material for: Assessment of the Efficacy of a Low-Dose Iron Supplement in Restoring Iron Levels to Normal Range among Healthy Premenopausal Women with Iron Deficiency without Anemia
Source: Nutrients. 2023 Jun 3;15(11):2620. doi: 10.3390/nu15112620 (PMC10255632; doi:10.3390/nu15112620)
Supplement: Supplementary file 1 [file nutrients-15-02620-s001.zip › nutrients-2408286-supplementary.pdf]

Study Eligibility Criteria.

Inclusion Criteria

- Healthy female participants > 18 years old
- Premenopausal (experiencing regular menstrual cycles)
- Hemoglobin > 11.0 g/dL
- Serum ferritin < 70 ng/mL
- C-reactive protein (CRP) < 3 mg/L
- BMI 18-29 kg/m^2
- No intake of dietary supplements containing iron 30 days prior to enrollment
- Willing and able to give written informed consent
- Able to read, understand, sign and date the informed consent document (English only)
- Able and willing to comply with the schedule visit(s) and study requirements

Exclusion Criteria

- Currently taking (within the past 14 days) dietary supplements including, vitamins (any), minerals, protein shakes, vitamin water, other supplements
- History of gastrointestinal disorders that could lead to uncertain absorption of the study supplements, (i.e., inflammatory bowel disease, ulcerative colitis, Crohn's disease, colostomy, or eating disorder)
- History or current malignancy
- Receiving chemotherapy agents or radiation treatments
- Prior health issues showing high CRP or other inflammatory markers
- Pregnancy or has breast fed within 3 months prior to enrollment
- BMI <18 or >30 kg/m^2
- Diagnosis of a terminal illness
- Use of prescription medications that impact digestion (i.e., proton pump inhibitor medications, other)
- History of alcohol abuse
- History or current drug abuse
- History or current cigarette smoke (including vaping) within the past 14 days from the screening visit
- Insulin-dependent diabetes and/or metformin use
- Chronic kidney or liver disease
- Anemia
- Has significant concurrent illnesses (controlled or uncontrolled), such as diabetes, lupus, epilepsy, or cardiac disorders, hepatitis B/C, HIV, serious mental health illness such as dementia or schizophrenia; psychiatric hospitalization in the past two years, or other, which in the opinion of the investigator, such condition might be aggravated because of treatment
- The investigator feels that for any reason the participant is not eligible to participate in the study
- History of uncontrolled cardiovascular disease (i.e., myocardial infarction, hypertension, hypercholesterolemia, peripheral vascular disease, other)
- History of or current bleeding disorders (i.e., hemophilia or von Willebrand disease), or anticipated treatment with prescription anticoagulants
- Developmental disability or cognitive impairment that would preclude adequate comprehension of the informed consent form and/or ability to follow study participant requirement and/or record the necessary study measurements
- A family member of the investigator or an employee of the investigator
- Participation in any other investigational study within 30 days prior to consent
